# The Beginning of More Worries: Doctoral Candidates’ Untold Stories After Submission of Dissertation

**DOI:** 10.3389/fpsyg.2020.537366

**Published:** 2021-01-15

**Authors:** Syed Abdul Waheed, Nadia Gilani, Mehwish Raza, Farooq Ahmad

**Affiliations:** ^1^Department of Educational Research and Assessment, Faculty of Education, University of Okara, Okara, Pakistan; ^2^Department of Teacher Education, Faculty of Education, University of Okara, Okara, Pakistan; ^3^Faculty of Education, Forman Christian College (A Chartered University), Lahore, Pakistan; ^4^Federal Government Educational Institution, Okara Cantt, Okara, Pakistan

**Keywords:** doctoral candidate, review report, thesis submission, narrative, Ph.D. delay

## Abstract

The present study focused on this particular situation in which doctoral candidates become anxious, impatient, and disappointed while experiencing a prolonged delay in processing their dissertation during and after the submission. The researchers tend to explore doctoral candidates’ storied experiences they had while confronting such procedural barriers and delays. We undertook a narrative mode of inquiry to explore the events and storied experiences through interviewing doctoral candidates from public universities in the province of Punjab, Pakistan. Nine doctoral candidates were selected through snowball sampling with the criterion of including those participants who were waiting for their external reviews at least for more than 1 year. From the narratives, the emergent themes include supervisors’ mutual relationships, the pressure of paper publication, lack of administrative support, external evaluation and follow-up and stress of delayed evaluation. The study has implications for relaxing procedural formalities during and after submission of a doctoral dissertation to facilitate students in the timely attainment of their doctoral degrees.

## Introduction

Recognizing the importance of research output, countries all over the world are committing considerable resources in order to bring advancement in their societies. In this regard, substantial investment is being made on doctoral candidates who are contributing significantly to the development of society (van de Schoot et al., 2013; Falk et al., 2019). The institutions of higher education provide opportunities for research and address students’ needs to accomplish their studies. They play a vital role in shaping doctoral students’ future as academic practitioners, early career researchers, and critical thinkers.

The achievement of a doctoral degree is considered one of the most significant educational fulfillments of students by most of the leading academic institutions and is viewed as the intellectual property and asset (Park, 2005; Jairam and Kahl, 2012). The education and professional development of doctoral students as researchers is one of the essential functions of most leading educational institutions of the world as these institutions of higher education offer their services for the socio-economic development of the country (Maqsood et al., 2019). In the same way, Millett and Nettles (2006) argue that through doctoral education, future faculty are trained, and the development of future educational leaders is carried out. Doctoral students intend to create new ideas and knowledge on which future educational policies can be built, preserved, and fed (Davis et al., 2006). Besides, doctoral students are the UN armed forces for the exchange of ideas between universities, nations, and all stakeholders (Thune, 2009).

In a tremendously changing world of today, research has become one of the most fundamental academic property rights for everyone to change their lifestyles according to their requirements (Maqsood et al., 2019). Mostly, doctoral students are given 3–4 years for completing their degrees by the universities, but on the average, they take more than the given time (Sadlak, 2004). Doctoral candidates often experience problems and challenges that not only delay their studies but prevent the accomplishment of their dissertation. From earlier research, it is evident that the students face many problems at the doctoral level, and there are complementary factors that contribute to this scenario (Wright, 2003). Such research shows that students, across the globe, find difficulties in earning degrees, and it often takes much time to complete their doctorate. Many of them fail to complete at all (Canadian Association for Graduate Studies, 2006). Feldon et al. (2010) also stressed that delay at the final stage in completing a doctoral degree could be very damaging for the students. It may lead them to terminate their studies, which cost a lot in terms of wastage of time, money, resources and guidance and ultimately severely affected the well-being of doctoral candidates (Bourke et al., 2004).

It has been noticed that doctoral students who remain tense and feel anxiety throughout their doctoral journey become impatient after the submission of their dissertations and anxiously wait for the day when they can receive the evaluation reports from the external evaluators and appear for the defense. This phenomenon is very common in countries where the responsibility is not much realized, and certain matters in the doctoral process are not prioritized. In this regard, there is not much research found in the context of Asia, particularly in Pakistan, while in a European perspective, doctoral students’ supervision is considered as much investigated field on serious grounds (Sidhu et al., 2014). Keeping in view the background of this significant problem of getting very delayed review reports from the external examiners and appearing in the public defense at a very later stage, the researchers have attempted to collect the perspectives of doctoral students based on the experiences they had with the supervisors and who have been anxiously waiting for their doctoral public defense. The researchers intend to carry out this study on finding the problems and challenges of doctoral students after submission of the dissertation to a supervisor, waiting for external evaluators’ feedback, and to have grasped the nature of the response, feelings, and state of anxiety doctoral students experience during this process.

## Context of Doctoral Studies

**FIGURE 1:**
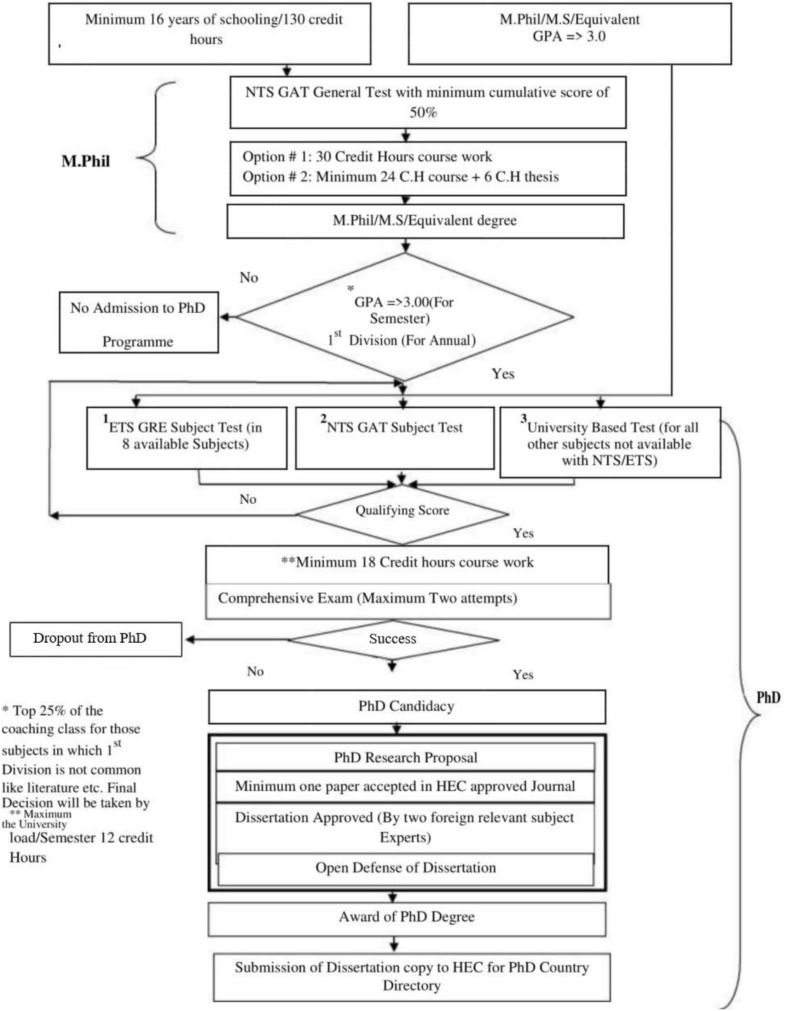
Process and requirements for completion of Ph.D. Source: Higher Education Commission of Pakistan (2017).

The doctoral studies organized in Pakistani universities are structured and guided by Higher Education Commission of Pakistan (HEC). The Commission has disseminated these guidelines to all public and private universities and has advised that the Degree Awarding Institutions must meet the minimum criteria for recognition of the degree by the HEC (HEC, 2017). The information about the process of doctoral studies and a clear description of the requirements for conferral of degree has been given in Figure 1.

In Pakistan, the doctoral degree is conferred upon the successful completion of course work of at least 18 Credit Hours followed by a comprehensive examination in most of the universities and after a successful evaluation and defense of the thesis. Typically, there is only one supervisor assigned to doctoral candidates. Nevertheless, doctoral candidates may be assigned a second supervisor as per the university rules and the dissertation’s requirement. The submission procedures are generally communicated to the doctoral candidates through a prospectus, the university’s website, and by department’s head. The set of rules are available at the time of admission to all the candidates. Most of these procedures are common in public and private universities in Pakistan. The data on the number of Ph.D. candidates in Pakistan was not found. The present study was conducted on the doctoral students enrolled in physical and social sciences in Pakistani universities.

## Purpose of the Study

The purpose of the research is to seek an understanding of doctoral candidates of how do they view the process of submitting their dissertations and waiting for an extended period and how do they experience and respond to the unnecessary delay in reaching the dissertation review reports very late. It also attempts to explore experiencing further delays caused by department faculty and administration due to procedural obstacles and non-cooperation of the university administration. The study purports to explore doctoral candidates’ perspectives and experiences on their doctorate being delayed and the role of the university in this process.

## Research Questions

The researchers intended to address the following research questions:

1. What do doctoral candidates who have experienced significant delays narrate about the practices of submission and review of the dissertation?
2. How do doctoral candidates who have experienced significant delays experience institutional support before and after the submission of the dissertation?
3. What are doctoral candidates’ narratives about the extraordinary delay of review reports and how do they respond to this situation?

## Mode of Inquiry

The researchers from higher education and research organizations are increasingly employing a qualitative mode of inquiry for exploring experiences of doctoral students, faculty, and such other study participants. Narrative inquiry, a form of qualitative research, was used in the present study that aims to examine the in-depth understanding of the meaning participants assigns to their storied experiences. Such studies are conducted on a small group of people to gather rich and free-ranging discourse (Salkind, 2010). Anderson and Anderson (2012) argued that narrative research “most often appear as highly personalized in-depth case studies of individual participants” (p. 241). Doctoral candidates’ experiences of submission of theses and delayed review process were considered as the “storied experiences” that reflected diverse narratives leading to discovering of commonalities of participants’ understanding of their experiences as “candidates waiting for their theses.” The participants’ experience of anxiety during waiting for receiving external evaluation reports and preparing for the defense of the dissertation was described through the events that occurred during the delaying period and stories they had with relevant persons of faculty and administration offices.

### Recruitment of Participants

Most of the participants in this study were independent researchers who were not employed in a research project. All of them were based in the province of Punjab in Pakistan. The researchers interviewed nine Ph.D. candidates who were selected with a purpose to gain an in-depth understanding of the storied experiences and perspectives of being their theses extraordinarily delayed after submission for evaluation (Creswell, 2009; Merriam, 2009). Those doctoral candidates were selected who had specific profiles as a Ph.D. candidate and had been waiting to receive external evaluation reports of their dissertations for at least 1 year, and they desperately wished to defend their dissertation at the earliest opportunity. In order to grasp extensive data, the participants were selected from different disciplines in public universities to allow maximum variation in their perspectives (Lincoln and Guba, 1985). Snowball sampling was used to identify a specific group of participants. In terms of their disciplines, five were selected from different fields of social sciences and four from various fields of physical science. Regarding the financial support and type of funding, three participants received a research grant, one was holding a regular position of research assistant, and five participants were self-financed. There were two female and seven male doctoral candidates who participated in the study.

Typically, fewer females are enrolled as doctoral students, as most of them get married after graduation. On the contrary, females studying in the undergraduate program seem to be more in number in most undergraduate programs. The study’s primary focus was not to find the disparity among the male and female doctoral candidates’ experiences of significant delay in doctoral conferral. The study was designed to explore the narratives of doctoral candidates about the delay phenomenon.

### Data Collection and Analysis

Consistence with the qualitative nature of the narrative inquiry, the researchers employed interviewing as a method of data collection. A focus group discussion did not seem possible as the participants of the study were scattered and could not be approached collectively. Semi-structured questions were framed to address the research questions of the study (Vekkaila et al., 2012). The interviews were undertaken until the point where: (1) the participants reflected the common and diverse experiences and perspectives of extraordinary delay in receiving the doctoral evaluation reports and waiting for the public defense their dissertation; and (2) adding new participants offered little new information (Creswell, 2007). We kept interviewing new participants until they came to the point where they met the purpose of the research, and various aspects of the phenomenon were revealed. This series of interviews continued until the new information was ended to emerge. Supporting this idea, Mason (2010) explained that this is “the point at which it becomes counterproductive and that the new that is discovered does not necessarily add anything to the history, model, theory or general framework” (p. 25). In addition to the interview, doctoral candidates were asked certain short questions that reflected their characteristics as research students such as age, gender, research experiences, the status of employment, working in a research project, or independently and discipline of study. Some of the characteristics emerged during the interviewing, and these were noted and analyzed.

The process of qualitative analysis began at the point of informal interaction and gaining insights into participants’ characteristics in association with the phenomenon under investigation (Patton, 1980; Rubin and Rubin, 2005; Merriam, 2009). The participants’ stories unveiled many aspects of the delaying the completion that helped to understand and analyze the interview transcripts. Field notes were taken and subsequently analyzed along with the interview text. The interviews were personally transcribed to allow us to immerse with the data and share the understanding of participants’ perspectives (Creswell, 2009). Each transcript was read several times and coded, compared continuously, and classified thematically into naturally emerging themes. The sufficient and substantive data emerged during interviews informed the development of core categories and their properties (Corbin and Strauss, 2008; Creswell, 2009).

## Results

The participants of the study seemed anxious and disappointed by the way their thesis submission and evaluation process proceeded. They were eager to share and discuss the issues and reasons for the delay in submission and waiting for the evaluation of their doctoral dissertation. The challenges emerged after interviewing the participants of the study are: supervisors’ mutual relationship and their supervisees, pressure of requirement of publication of the paper, lack of administrative support from head of the department, dean of the faculty and the controller of examination, inappropriate communication through electronic and internet sources, external evaluation and inactive follow-up mechanism by the administrative departments and a state of stress caused by the extraordinary delay. The participants’ experiences under each theme are discussed below.

### Supervisors’ Mutual Relationship

Professional jealousy exists in almost every profession and every culture (Dammani, 2019). It can be positive if taken as just competing others, not destroying them. In the teaching profession, it becomes even more critical as in most cases; teachers target not only the other teacher but also his students. At the university level, the doctoral students need the support of other faculty members, along with his supervisor, to carry out their research smoothly.

The study participants reflected that professional jealousy among the faculty members and their associated staff was found to be very common in the concerned departments of universities. The participants were of the view that research supervisors did not allow their doctoral candidates to meet them or sit in their offices. They could not take assistance from other professors in faculty even sometimes they needed desperately due to the expertise of those professors in related areas of their research. One of the participants in the study revealed that:

Once on my way to my supervisor’s classroom, I met one professor who was not in the “good books” of my supervisor. I greeted him and stayed for a few minutes with him. My professor saw me from the classroom and became so annoyed that my supervisor did not let me come to his class on that day.

The participants stated that this professional jealousy was also one of the causes in delaying not only the research process but even the submission and evaluation process as well. Those faculty members who were close to the members of the final approval committee propagated unnecessarily regarding the dissertation and tried to slow down the process of submission. They did not want their colleagues to get credit for supervising a doctoral dissertation that is considered *“a jewel in the crown”* for a faculty member in the university. One of the doctoral candidates sadly described that when I was about to submit my thesis, I got to know from one of my class fellows that his supervisor who was not in good terms with my supervisor told him that *“I will see how he would be able to submit his thesis.”* His statement made me devastated and left me in shock.

### Pressure of Paper Publication

Publishing one or two research papers in journals recognized by the Higher Education Commission of Pakistan (HEC, is one of the requirements of doctoral candidates to accomplish their doctoral degrees and finally submit to the department. It proved to be one of the significant causes of delay in the submission process as no candidate can submit his/her thesis without publishing the required number of articles in a journal.

Most of the participants reflected on this condition of publishing articles that it made us suffered a lot. Publishing in a journal is a time taking process and in most of the cases, it bears a high cost. Generally, universities do not have their journals indexed by HEC. This is the reason why candidates have to search for other national or international journals to get their research papers published related to their doctoral research. This situation created fierce competition. A race has started for getting the papers published in the journals for fulfilling HEC criteria and candidates have to wait in a long queue of papers. Most of the participants expressed their disapproval of this condition as it became a serious issue in the delay of submitting their research work. One of the participants stated, “*where we would go for the publication*. *No one accepts our paper to publish.*” Another participant aggressively remarked:

Why these journals are doing this with us. They are taking undue advantage of our vulnerable condition. How much we have to wait even after completing our research task. I wish I could have my own journal so that I could complete my dissertation in time.

Some of the HEC recognized journals have high processing fees for publishing a research paper, especially those who have more issues. One of the participants who was financing his studies himself remarked that “*international journals demand money to publish research a paper whereas I hardly manage the expenses of my studies. From where I should bring a bundle of rupees to fulfill this requirement of publishers*.” He elaborated that he had to wait for months in collecting the required money even after completing his research, but the problem did not end here. Journals are so overloaded that despite giving processing fee, he waited for another year to get his paper published.

### Lack of Administrative Support

Where the doctoral candidates need support from their supervisor, they equally need assistance from the administrative staff of the department and university as well. It includes the Dean of Faculty, Head of Department (HOD), Board of Advanced Studies and Research (BASR) and Quality Enhancement Cell (QEC). They all have to play their part at the different stages of thesis completion and submission. Faculty members guide them in various steps of their research process and administrative staff helps them attaining their final degrees.

It was noticed from the participants’ stories that they experienced a lack of support from the academic and non-academic staff of the university that resulted in a significant delay in sending the theses and receiving the evaluation reports back. They had varied experiences and showed different responses to such a lack of support. The doctoral candidates complained that the university administration usually shows a lack of responsibility, which causes a delay in submission and sending dissertations abroad for external evaluation. One of the doctoral candidates shared that “*Surprisingly, universities work only for their financial benefits and do not care about the interests of their students*. *Universities enroll a high number of Ph.D. candidates without keeping in view the resources available and their limitations.”* He explained that as one supervisor can have a limited number of supervisees at a time, the administration used delaying strategy by keeping the completed work in the queue unless a place for a student is created by clearing already submitted thesis.

Another participant in the study shared his experience regarding the lack of support from the QEC. He told that after submitting a thesis in my department, it went to QEC for taking the plagiarism report and to assess whether all the requirements have been fulfilled for the submission. They took almost three to 4 months to complete the documentation process that needs a few weeks and yet they were waiting to forward it to external supervisors. Upon asking twice about the status of his thesis, one of the officials in the QEC got furious by saying:

We have many tasks to do other than this, so do not come again and again. Why you are in so much hurry to become a doctor. He further threatened me by saying that if I kept on coming like this, he would further delay the documentation process.

According to another doctoral candidate, “no one is ready to take responsibility to solve doctoral students’ issues of delay and timely processing of their dissertations.” One of the participants complained that “*there is no check and balance for administrative and student supporting staff. They are too careless. They never bother to handle issues. Everyone says in Pakistan that it is not my duty.*” Most of the participants had similar experiences regarding administrative support. In their views, they need to be supported by the personnel as they reached this stage after facing a lot of hardships and struggle in carrying out the research process.

### External Evaluation and Follows-Up

Another condition of completing the degree of a doctorate is the positive external evaluation report not only from national/local evaluator but also from two foreign evaluators. Based on these reports, public defense is arranged by the department. Most of the doctoral candidates found this step the most challenging one. There are many reasons for this. According to the participants, in few cases, they had to give the names of local or foreign evaluators, but they do not know many of them due to the limited or no interaction with the experts of their area other than their own teachers. They are dependent on their supervisors to find out the relevant local and foreign evaluators. One of the participants shared his experience of selecting a foreign evaluator that *“I am the first Ph.D. candidate of my supervisor and my supervisor instructed me to find out the foreign evaluator by myself. How can I approach to a foreign evaluator if my supervisor cannot?*”

Worries did not end here; “*in many cases, the supervisor has to manage to find a foreign evaluator*,” another participant added. After finalizing the names of external evaluators, the university administration kept students’ theses for months without any justification. On pursuing again and again, when dissertations are sent to them, there is no proper follow-up mechanism for getting the evaluation reports back in time. For example, in the case of a study participant, she explained:

From the department, my thesis was forwarded to the foreign evaluators through air-mail, but they did not confirm from the evaluators. Almost 5 months had gone, and nothing came back from them. Upon asking again and again from the department, I always received the answer, “wait.” After 5 months of wait, it was discovered that the evaluators did not get my thesis.

Another aspect of not getting evaluation reports in time was that the external evaluators did not get paid by the university administration timely. University charged half of the evaluation fees from the doctoral candidates and half of it is to be paid by the university itself. One of the participants remarked that:

How much more money they require from us? We are even ready to pay the charges which university has to pay, but we want to come out of this phase of high anxiety and tension caused by the unnecessary delay in our degree completion.

Due to all these aspects of the delay, doctoral candidates became hopeless and imagined that they would be able to complete their degrees. Another participant added that “*the academic and non-academic staff is very irresponsible and they do not understand our problems. They feel that we are a burden for them.”* Similarly, one of the participants was much hopeless and reflected that *“We do not see any hope from anywhere. How much patient can we be? It is beyond our limits.”*

One of the aspects of delay and barrier in following the review process and coordination with faculty of the university and examination department was revealed when the participants of the study disclosed that their research supervisor had a poor understanding to use internet sources, mainly the use of the internet, software, and e-mails. They only knew the basic applications of MS Word and they were not acquainted with other sources; for example, they could not send e-mails and contact the concerned persons to get feedback on a doctoral thesis. One of the doctoral candidates tried to coach his supervisor to use such sources of communication and he refused to comment that he was too old to learn such things and he would not be able to understand it. As a result, doctoral candidates had to contact the university administration directly but their inappropriate behaviors increased ambiguity and worries of the delay. Some of the candidates remarked that “*my research supervisor cannot use a computer,”* and *“it’s a pity he cannot use modern technology for everyday needs*.” The participants described that they might feel that they are near to retirement, so learning to use the internet would no longer be useful in their careers. One of the participants added that he tried to convince his supervisor on using technology but he stated that “*I can’t handle your technological gadgets*.”

### Stress of Delayed Evaluation

Stress is a common phenomenon associated with doctoral studies and students. Maintaining mental health is a challenging task, especially for doctoral students who are working continuously in a challenging environment. According to Stubb et al. (2012), doctoral studies have a negative impact on the well-being of Ph.D. students and its level varies from discipline to discipline, university to university, supervisor to supervisor and student to student under various conditions and circumstances. Many doctoral students think of even leaving their Ph.D. studies at some point due to the massive effect on their mental stability (Anttila et al., 2015).

According to the participants of the study, they found themselves in high stress due to the undue delay in accomplishing their doctoral degrees. This was a delay on account of unnecessary procedural formalities undertaken by the official of the department. Not only doctoral candidates but their family members and relatives were affected mainly by this process. They kept on asking them, *“when your degree will be finished and when you are going to become a doctor.”* On having no answer to this question and feeling helpless, they experienced health issues like headaches, stomach aches, muscle stiffening.

One of the female participants shared her mental condition that she was so disturbed due to the delay in her evaluation process that *“all the time she only thought of getting back her evaluation reports even in her dreams.”* This situation made her suffer from extreme stress and anxiety. Another female participant having children, also suffered from a high level of mental stress. She described that after the long journey of conducting an exhaustive research process, she thought her struggle was over now, but she did not know that many phases of hardships like submission, evaluation and defense were waiting for her.

In some cases, the stress went to another level and got converted into diseases. A couple of the doctoral students who were in their fifties stated:

This wait for receiving the external evolution report has made us patients of high blood pressure and diabetes. These obstacles that cause a delay in our doctoral degree completion would cost us for a life as it is killing for us internally due to massive pressure.

Almost all the participants of the study experienced extreme stress associated with the unusual and unjust delay in their evaluation process that affected their mental health badly.

## Discussion

The purpose of this study was to explore the narrative perspectives of doctoral candidates at the time of submission and during the evaluation process of their dissertation. After analyzing the themes that emerged from participants’ interviews, it was found that most of the research participants have not had pleasant experiences regarding submitting their doctoral dissertation and during the evaluation process. They kept on waiting for a long period to be able to submit their dissertations and get back evaluation reports finally.

There were multi-dimensional aspects of the barriers and delays during and after submission. One of them was professional jealousy and non-cooperation among the supervisors within departments. Out of this, delaying tactics were used by them, not let them take credit for research, which eventually causes sufferings for the supervisor and his/her supervisee. The university administration should take serious action of reporting any of this delaying tactic to smooth proceedings of completed work of the candidate. Moreover, personal grudge between members of the faculty of the same department and lack of interest in other doctoral candidates’ research due to personal conflicts with their supervisors, cause many hindrances for proper processing of dissertation work (Helm, 1989; Jacobs, 1994; Johnston et al., 2016; Katz, 1997; Sayed et al., 1998; Mouton, 2001). The other researchers emphasized that the successful completion of a dissertation is acutely dependent on collaboration between the candidate and the research supervisor for fair judgment of abilities of a candidate and smooth process of the dissertation after completion (Smith et al., 1993; Hockey, 1994; Rademeyer, 1994).

Another hurdle that caused a delay in the submission was the requirement of a research paper published in the Higher Education Commission (HEC) recognized journal, which became a real headache for the doctoral candidates. There was a considerable number of papers to be published by the journals, so they take a long time as well as heavy processing fees, which wastes candidate’s lot of time in waiting to get their work published, and most of them could not bear the enormous publication fee. Departments should launch their journals fulfilling HEC criteria, and doctoral candidates’ work should be published on a priority basis for timely submission of their work.

Lack of administrative support was another important aspect of late submission and completing the evaluation process in time. Quality Enhancement Cell (QEC) needs to work within the given period to complete all the procedural and documentation requirements to avoid any delay in completing a doctoral degree. The role of administration of the department and supervisors’ in the entire process of thesis evaluation is an essential factor (Barnes et al., 2010). Therefore, the evaluation process and fair judgment can be of varying types according to the nature of the field, the supervisor’s attitude, and the working conditions. According to Zuber-Skerritt and Roche (2004), generally speaking, it is expected that the doctoral candidates demand not only support, time and encouragement, but also resources and information, feedback and after submission of dissertation the contact to the external evaluator for evaluation reports and the defense of a dissertation on behalf of doctoral supervisor. Authenticity and communication (during the whole process of evaluation) should be his possessions.

The role of research supervisor, nonetheless, is that of fundamental value for the refinement of doctoral candidates’ abilities to work productively and their supervisors would be playing vital role in their respective fields of research in academia, and this gives a specific recognition and identity to the doctoral candidates as well (Barnes et al., 2010; Halse and Malfroy, 2010; McAlpine and Amundsen, 2012). Then comes the mutual understanding of supervisors and the institutional administration on the basic issues emerging as a result of doctoral candidates’ and supervisors’ mutual relationships and those about other researchers and the research process.

Moreover, there should be a proper mechanism to find and access the relevant external evaluators. University departments have a list of professors/evaluators along with their consent in every relevant field to save the candidates or their supervisors from the hassle of searching them. Most of the doctoral candidates’ time is gone wasted in doing this practice. After sending dissertations to external evaluators, including local and foreign evaluators, confirmation calls and emails should be made to make sure that dissertations have reached to them. Above all, they should be paid on time to make the evaluation process fast. According to Helm (1989), the problems are 3-fold, namely; problems in the selection of appropriate external evaluators, the submission of the dissertation and receiving the evaluation report in due time. He remarked that these problems could be due to insufficient collaboration between institutional management and external evaluators, poor connection, and the functioning of the system. All these factors contribute to unwanted delays in completing a doctoral dissertation and make candidates suffer from extreme stress and anxiety in turn. They lost their family and social life due to the enormous pressure of quickly accomplishing the Ph.D. task from them and get isolated. This isolation leads them toward some severe illness.

### Implications and Recommendations

The results of this study highlight the different problems and challenges being faced by the doctoral candidates during and after the submission process of the dissertation and tend to evoke the concerning university authorities to address the situation appropriately. The study has implications for the improvement of this process by involving the management and faculty of the relevant department and designing an effective policy for the efficient mechanism of the submission process and acceleration of the review process. The study addresses all the stakeholders of the higher education institution to facilitate doctoral candidates at the end of this tedious journey of scholarly pursuits. It could be highly profitable, especially in the context of the education system of Pakistan at a higher level. The universities and Higher Education Commission of Pakistan (HEC) may take serious steps to lessen the severity and “tragedy” of the state of “waiting to reach the destination of doctorate.” This study is a source of information to the candidates, faculty, administration and external evaluator about one of the most critical issues emerging in the life of doctoral candidates and it would also encourage and motivate the candidates to continue their higher studies without the fear of hindrances during the process of doctorate, and they would be able to work with the best of their efforts. Moreover, “in order to participate successfully in this transformed global context, national Ph.D. education programs need not only to be excellent, efficient, and transparent but also to be recognized as such internationally” (Nerad and Evans, 2014, p. 1). Also, “doctoral education worldwide is converging in the sense that top national or regional flagship programs now have similar principles and structures, resulting in an intensified stratification of doctoral education training, and bringing about new tensions in doctoral education” (Nerad, 2020).

The researchers got an opportunity to be very close to what the participants of the study felt that helped understand the phenomenon. The emotions, anxiety, passion, desperation, and disappointment experienced by the doctoral candidates is what requires for certain recommendations for facilitation and guidance. It demands further exploration as this broad research area has not yet been sufficiently studied. There are not many theories found in the literature that can organize all that is known and explored so far about the doctoral candidates’ experiences after submission of the dissertation. The development of such a theory or model would deepen and critically appraise the accessible knowledge in the field through a vigorous exploration of executing qualitative research. Further studies may be conducted by using a mixed-methods design with the participants from the other disciplines of the science, arts, humanities, and social science to grasp a broader perspective of the phenomenon.

## Conclusion

Doctoral candidates’ narrative perspectives reflected that professional jealousy among supervisors and their colleagues that was targeted at disowning credit of research to others, pressure of publishing a research paper out of the dissertation on a fast track, lack of administrative support in timely submission and forwarding the dissertation to external evaluators and not following them up were some of the aspects of the delay and barriers during and after submission of doctoral dissertation that led the participants in a state stress, anxiety, and disappointment. These results imply that doctoral candidates realize a state of “undone” when they had done all that they need to do. It is required that an effective and efficient support mechanism should be devised to facilitate doctoral candidates for timely completion after they have completed the dissertation.

## Data Availability Statement

All datasets generated for this study are included in the article/supplementary material.

## Ethics Statement

The studies involving human participants were reviewed and approved by the Ethical Research Committee of University of Okara, Pakistan. The patients/participants provided their written informed consent to participate in this study.

## Author Contributions

SAW, NG, and MR conceived of the idea, worked on research design, critically reviewed and revised the execution of the study and the relevant ethical considerations, conducted additional interviews to saturate the data, analyzed the interview transcripts and finalized the draft for submission. FA gathered the initial data, conducted a preliminary analysis, and outlined major parts of the manuscript. All authors contributed to the article and approved the submitted version.

## Conflict of Interest

The authors declare that the research was conducted in the absence of any commercial or financial relationships that could be construed as a potential conflict of interest.

## References

[B1] Anderson S. Anderson B. (2012). Preparation and socialization of the education professoriate: Narratives of doctoral student-instructors. *Int. J. Teach. Learn. High. Educ.* 24 239–251.

[B2] Anttila H. Lindblom-Ylänne S. Lonka K. Pyhältö K. (2015). The added value of a PhD in medicine-PhD Students’ perceptions of acquired competences. *Int. J. High. Educ.* 4:172.

[B3] Barnes B. J. Williams E. A. Archer S. A. (2010). Characteristics that matter most: Doctoral students’ perceptions of positive and negative advisor attributes. *NACADA J.* 30 34–46. 10.12930/0271-9517-30.1.34

[B4] Bourke A. Holbrook A. Lovat R. Farley P. (2004). “Attrition, completion and completion times of PhD candidates,” in *Procceding of the AARE Annual Conference Proceedings*, (Melbourne).

[B5] Canadian Association for Graduate Studies. (2006). *Graduate Studies Review Report. 301-260 St.* Ottowa: Patrict Street, 172–188. 10.1080/15348431003761166

[B6] Corbin J. Strauss A. (2008). *Basics of qualitative research: Techniques and procedures for developing grounded theory*, 3rd Edn Thousand Oaks, CA: Sage.

[B7] Creswell J. W. (2007). Five qualitative approaches to inquiry. *Qual. Inq. Res. Des. Choos. Five Approaches* 2 53–80.

[B8] Creswell J. W. (2009). *Research Design: Qualitative, Quantitative, and Mixed Methods Approaches*, 3rd Edn Thousand Oaks, CA: Sage.

[B9] Dammani K. (2019). *A study of the professional jealousy among teachers.* Munich: GRIN Verlag.

[B10] Davis H. Evans T. Hickey C. (2006). A knowledge-based economy landscape: Implications for tertiary education and research training in Australia. *J. High. Edu. Policy Manag.* 28 231–244. 10.1080/13600800600979983

[B11] Falk L. L. Augustin H. Torén K. (2019). Doctoral students’ perceived working environment, obstacles and opportunities at a Swedish medical faculty: A qualitative study. *BMC Med. Educ.* 19:250. 10.1186/s12909-019-1684-x 31286962 PMC6615109

[B12] Feldon D. F. Maher M. A. Timmerman B. E. (2010). Performance-based data in the study of STEM PhD education. *Science* 329 282–283. 10.1126/science.1191269 20647452

[B13] Halse C. Malfroy J. (2010). Retheorizing doctoral supervision as professional work. *Stud. High. Educ.* 35 79–92. 10.1086/603932

[B14] HEC (2017). *Minimum Criteria for MS/M.Phil and Ph.D. Programs.* Retrieved from https://www.hec.gov.pk/english/scholarshipsgrants/Documents/MPHIL_Phd_Criteria.pdf

[B15] Helm C. A. Q. (1989). Maatreëls om die probleme van nagraadse navorsingstudente te verminder - ’n literatuurstudie. *Suid-Afrikaanse Tydskrif vir Hoër Onderwys* 3 79–85.

[B16] Hockey J. (1994). Establishing boundaries: problems and solutions in managing the PhD supervisor’s role. *Cambridge J. Educ.* 24 293–305. 10.1080/0305764940240211

[B17] Jacobs L. J. (1994). The role of the supervisor or promoter. *Progressio* 16 29–34.

[B18] Jairam D. Kahl J. D. H. (2012). Navigating the doctoral experience: The role of social support in successful degree completion. *Int. J. Doc. Stud.* 7 311–329. 10.28945/1700

[B19] Johnston L. Wilson T. Mackenzie A. (2016). Assisting Ph.D. completion following a natural disaster. *Int. J. Dr. Stud.* 11 367–382. 10.28945/3590

[B20] Katz E. L. (1997). Key players in the dissertation process. *N. Direct. High. Educ.* 25 16–19.

[B21] Lincoln Y. Guba E. (1985). *Naturalistic Enquiry.* Beverly Hills, CA: Sage.

[B22] Maqsood Z. Jabeen S. H. Chaudary N. R. Sardar I. (2019). Attitude towards research of university students, A Multivariate analysis. *Pyrex J. Educ. Res. Rev.* 4 37–43.

[B23] Mason M. (2010). Sample size and saturation in PhD using qualitative interviews. *Forum Qual. Soc. Res.* 11 1–13.

[B24] McAlpine L. Amundsen C. (2012). Challenging the taken-for-granted: How research analysis might inform pedagogical practices and institutional policies related to doctoral education. *Stud. High. Educ.* 37 667–681.

[B25] Merriam S. B. (2009). *Qualitative research: A guide to design and implementation.* San Francisco: Jossey-Bass.

[B26] Millett C. M. Nettles M. T. (2006). Expanding and cultivating the Hispanic STEM doctoral workforce: Research on doctoral student experiences. *J. Hispanic High. Educ.* 5 258–287. 10.1177/1538192706287916

[B27] Mouton J. (2001). *How to succeed in your masters & doctoral studies.* Pretoria: Van Schaik.

[B28] Nerad M. (2020). *Governmental Innovation Policies, Globalisation, and Change in Doctoral Education Worldwide: Are Doctoral Programmes Converging? Trends and Tensions. In Structural and Institutional Transformations in Doctoral Education.* London: Palgrave Macmillan, 43–84.

[B29] Nerad M. Evans B. (eds) (2014). *Globalization and its impacts on the quality of PhD education: Forces and forms in doctoral education worldwide.* Berlin: Springer.

[B30] Park C. (2005). New variant PhD: The changing nature of the doctorate in the UK. *J. High. Educ. Policy Manag.* 27 189–207. 10.1080/13600800500120068

[B31] Patton M. Q. (1980). *Qualitative evaluation methods*, 2nd Edn Thousand Oaks, CA: Sage.

[B32] Rademeyer G. (1994). Thesis supervision: getting the genie out of the lamp. *South Afr. J. High. Educ.* 8 92–95.

[B33] Rubin H. J. Rubin I. S. (2005). *Qualitative Interviewing: The Art of Hearing Data*, 2nd Edn Thousand Oaks, CA: Sage.

[B34] Sadlak J. (2004). *Doctoral studies and qualifications in Europe and the United States and prospects.* Bucharest: UNESCO.

[B35] Salkind N. J. (2010). *Encyclopedia of research design.* Thousand Oaks, Calif: SAGE.

[B36] Sayed Y. Kruss G. Badat S. (1998). Students’ experience of postgraduate supervision at the University of the Western Cape. *J. Further High. Educ.* 22 275–285. 10.1080/0309877980220303

[B37] Sidhu G. K. Kaurb S. Fooka C. Y. Yunus F. W. (2014). Postgraduate supervision: Comparing student perspectives from Malaysia and the United Kingdom. *Procedia Soc. Behav. Sci.* 123 151–159. 10.1016/j.sbspro.2014.01.1409

[B38] Smith S. W. Brownell M. T. Simpson R. I. Deshler D. D. (1993). Successfully completing the dissertation: two reflections on the process. *Remed. Special Educ.* 14 53–60. 10.1177/074193259301400310

[B39] Stubb J. Pyhältö K. Lonka K. (2012). The experienced meaning of working with a PhD Thesis. *Scandinavian J. Educ. Res.* 56 439–456. 10.1080/00313831.2011.599422

[B40] Thune T. (2009). Doctoral students on the university-industry interface: A review of theliterature. *High. Educ.* 58 637–651. 10.1007/s10734-009-9214-0

[B41] van de Schoot R. Yerkes M. A. Mouw J. M. Sonneveld H. (2013). What took them so long? Explaining PhD delays among doctoral candidates. *PLoS One* 8:e68839. 10.1371/journal.pone.0068839 23935895 PMC3720867

[B42] Vekkaila J. Pyhältö K. Hakkarainen K. Keskinen J. Lonka K. (2012). Doctoral students’ key learning experiences in the natural sciences. *Int. J. Res. Dev.* 3 154–183. 10.1108/17597511311316991

[B43] Wright T. (2003). Postgraduate research students: people in context?. *Br. J. Guid. Counc.* 31 209–227. 10.1080/0306988031000102379

[B44] Zuber-Skerritt O. Roche V. (2004). A constructivist model for evaluating postgraduate supervision: A case study. *Q. Assurance Educ.* 12 82–93. 10.1108/09684880410536459

